# TRPV4-Dependent Epithelial Mechanoadaptation and Barrier Remodeling Mediate Sennoside-Induced Distal Colonic Motility

**DOI:** 10.3390/ijms27115057

**Published:** 2026-06-03

**Authors:** Yusuke Hara, Rei Kawashima, Shun Tamaki, Tatsunori Maekawa, Yuki I. Kawamura, Taizo Tsujimoto, Chika Kusano, Takafumi Ichikawa

**Affiliations:** 1 Department of Gastroenterology, Kitasato University Graduate School of Medical Sciences, Sagamihara 252-0374, Kanagawa, Japan; yhara@insti.kitasato-u.ac.jp (Y.H.); tsujimoto.taizo@kitasato-u.ac.jp (T.T.); c-kusano@kitasato-u.ac.jp (C.K.); 2 Department of Gastroenterology, Kitasato University School of Medicine, Sagamihara 252-0373, Kanagawa, Japan; 3 Department of Regulation Biochemistry, Kitasato University Graduate School of Medical Sciences, Sagamihara 252-0373, Kanagawa, Japan; tamaki.shun@kitasato-u.ac.jp (S.T.); maekawa@kitasato-u.ac.jp (T.M.); t.ichika@kitasato-u.ac.jp (T.I.); 4 Department of Biochemistry, Kitasato University School of Allied Health Sciences, Sagamihara 252-0373, Kanagawa, Japan; 5 Regenerative Medicine and Cell Design Research Facility, Kitasato University School of Allied Health Sciences, Sagamihara 252-0373, Kanagawa, Japan; 6 National Institute of Global Health and Medicine, Japan Institute for Health Security, Shinjuku 162-8655, Tokyo, Japan; kawamura.y@jihs.go.jp

**Keywords:** TRPV4, mechanotransduction, epithelial barrier, E-cadherin, colonic motility, sennoside

## Abstract

Improvement of bowel function is accompanied by increased luminal flow and altered epithelial mechanical forces, yet the underlying epithelial mechanisms remain unclear. We investigated whether enhanced luminal stimulation is associated with epithelial mechanotransduction and junctional remodeling during changes in colonic motility. Sennoside was orally administered at 4.8 mg/kg body weight to 5-week-old male BALB/cAJcl mice for 21 days to model increased luminal stimulation. Stool characteristics, fecal water content, Bristol Stool Form Scale scores, and segmental colonic motility were assessed. Expression of Muc2, inflammatory cytokines, Trpv4, and E-cadherin was quantified across colonic regions. In CT26 monolayers, mechanical stress was applied to evaluate transient receptor potential channel induction, E-cadherin redistribution, and transepithelial electrical resistance, and the effect of *Trpv4* knockdown. Sennoside softened stools, increased fecal water content (+18%) and Bristol scores (+57%), and enhanced distal colonic motility (+117%) without altering inflammatory cytokines. Trpv4 was selectively upregulated in the distal colon (3.3-fold). E-cadherin expression increased (2.5-fold) with junctional redistribution, whereas Muc2 decreased (−44%). In vitro, mechanical stress upregulated Trpv4 (2.5-fold), increased barrier resistance (+48%), and promoted E-cadherin assembly; these effects were augmented by sennoside and attenuated by *Trpv4* silencing. These findings suggest that epithelial responses involving TRPV4-associated mechanotransduction and junctional remodeling are associated with altered barrier-related properties and distal colonic functional changes, providing insight into an epithelial component of stimulant laxative action.

## 1. Introduction

Constipation is a common functional gastrointestinal disorder with a high global prevalence and is associated with reduced quality of life and an increased risk of systemic diseases [1,2,3,4,5]. Delayed intestinal transit is frequently accompanied by alterations in gut microbiota composition and impairment of epithelial barrier integrity [6]. These changes in the intestinal microenvironment may influence neural, immune, and metabolic signaling pathways [7]. Collectively, these observations support the concept that constipation represents not merely a clinical symptom but a pathophysiological process that affects intestinal homeostasis.

In addition to lifestyle modifications, pharmacologic agents are widely used for the management of constipation, among which sennoside is a commonly prescribed stimulant laxative [8]. Sennoside is metabolically activated by colonic microbiota into active metabolites, including rheinanthrone, thereby enhancing intestinal motility [9,10]. However, prolonged exposure has been reported to alter epithelial architecture and disrupt mucosal homeostasis [11,12,13]. Despite these observations, the molecular mechanisms by which changes in the luminal environment induce adaptive epithelial responses remain insufficiently characterized.

The intestinal epithelium functions not only as a passive barrier but also as a dynamic sensory interface capable of detecting mechanical and chemical stimuli within the lumen and transducing through defined molecular pathways. Transient receptor potential (TRP) channels serve as key mechanosensitive regulators of gastrointestinal sensory functions [14]. Similarly, mechanosensitive ion channels, such as Piezo proteins, convert mechanical forces into intracellular signaling cascades that modulate epithelial behavior [15,16]. Furthermore, adhesion complexes organized around E-cadherin undergo mechanoregulated remodeling and contribute to the maintenance of epithelial polarity and barrier integrity [16,17,18]. Together, these mechanotransductive and adhesion-associated pathways may mediate adaptive epithelial responses to alterations in the local mechanical environment. In this study, epithelial mechanoadaptation is defined as the process by which epithelial cells sense sustained mechanical cues and adjust their structural and functional properties in response to such stimulation.

However, it remains unclear how improvements in bowel motility, accompanied by changes in luminal mechanical and physical forces, influence epithelial mechanosensitive signaling and junctional organization.

Therefore, in this study, we employed sennoside administration as a model of enhanced luminal mechanical stimulation and performed a comprehensive morphological and molecular characterization of intestinal epithelial cells. By examining TRP channel-mediated mechanosensitivity, E-cadherin-associated junctional remodeling, and dynamic changes in epithelial barrier function, we sought to elucidate the molecular basis of epithelial adaptation underlying functional improvement of intestinal physiology.

## 2. Results

### 2.1. Increased Stool Water Content and Enhanced Distal Colonic Motility

Food intake, water consumption, and body weight were monitored during the 21-day sennoside administration period to evaluate the systemic effects of chronic intestinal stimulation. No significant differences in food intake or body weight were observed between the control and sennoside-treated groups. Water consumption decreased slightly following sennoside administration; however, the magnitude of this reduction was minimal (Figure S1).

**Figure 1:**
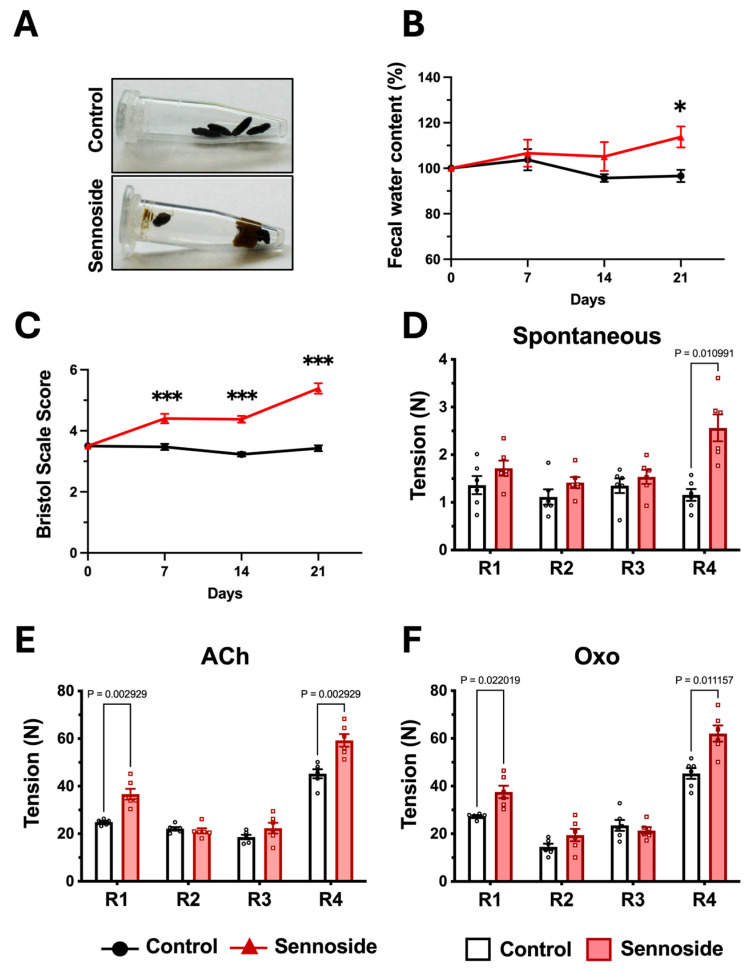
Fecal properties and colonic motility in sennoside-treated mice. Sennoside (4.8 mg/kg body weight) was orally administered once daily for 21 days. Representative fecal appearance (**A**), fecal water content (**B**), and Bristol Stool Form Scale (BSFS) scores (**C**) are shown. Spontaneous colonic motility was quantified using the Magnus method in four colonic regions on day 21 (**D**). Contractile responses to acetylcholine (ACh) and oxotremorine (Oxo) were measured (**E**,**F**). Data are presented as mean ± standard error of the mean. Each dot represents one mouse. * *p* < 0.05, *** *p* < 0.001. *n* = 15 mice per group for fecal analyses; *n* = 6 mice per group for motility analyses.

Macroscopic examination of feces on day 21 demonstrated visibly softer stool in the sennoside-treated group (Figure 1A). Quantitative analysis showed that fecal water content increased beginning on day 7 and reached approximately 18% above control levels by day 21 (Figure 1B). Assessment using the BSFS revealed a progressive increase in stool scores starting on day 7, with an approximately 1.5-fold increase by day 21 compared with controls (Figure 1C and Figure S2).

Colonic motility was evaluated ex vivo using a Magnus apparatus. Spontaneous contractile activity was increased throughout the colon in the sennoside-treated group (Figure S3A). When the colon was subdivided into four regions (R1–R4), as schematically defined in Supplementary Figure S4, the enhancement of spontaneous motility was most pronounced in region 4, corresponding to the rectum (Figure 1D and Figure S4).

To assess pharmacological responsiveness, isolated colonic segments were stimulated with ACh or Oxo. In control tissues, both ACh and Oxo elicited greater contractile responses in regions 1 and 4 than in regions 2 and 3. These contractile responses were further augmented following sennoside administration (Figure 1E,F and Figure S3B).

### 2.2. Noninflammatory Mucosal and Epithelial Alterations

**Figure 2:**
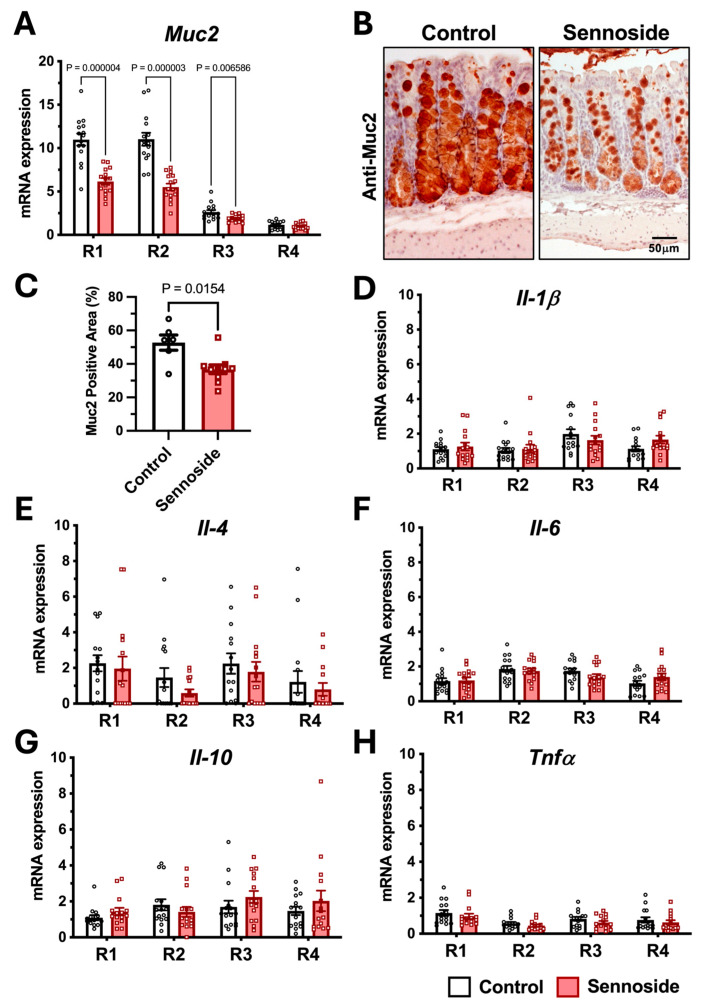
Mucin and cytokine expression in colonic tissues of sennoside-treated mice. *Muc2* mRNA expression was analyzed in four colonic regions on day 21 (**A**). Representative immunohistochemical staining for *Muc2* in Region 2, as defined in Figure S4, and quantification of positive areas are shown (**B**,**C**). mRNA expression levels of *Il1β*, *Il4*, *Il6*, *Il10*, and *Tnfα* were analyzed in each colonic region (**D**–**H**). Data are presented as mean ± standard error of the mean. Each dot represents one mouse. *n* = 15 mice per group for mRNA analyses; *n* = 6–10 mice per group for immunohistochemistry.

Mucin expression was examined across four colonic regions to characterize epithelial responses to sennoside administration. *Muc2* mRNA expression showed a decreasing trend in regions 1–3 in the sennoside-treated group (Figure 2A). Immunohistochemical analysis demonstrated reduced Muc2 protein expression in goblet cells following sennoside administration (Figure 2B,C). Muc1 expression was also reduced in region 1 (Figure S5).

Epithelial cell proliferation was evaluated using Ki-67 immunostaining. The number of Ki-67–positive epithelial cells was reduced in colonic tissues from sennoside-treated mice (Figure S6).

To assess inflammatory responses, mRNA expression levels of *Il1β*, *Il4*, *Il6*, *Il10*, and *Tnfα* were quantified in colonic tissues. No significant differences were observed between control and sennoside-treated groups in any colonic region (Figure 2D,H).

### 2.3. Distal Colonic Upregulation of Mechanosensitive TRP Channels

**Figure 3:**
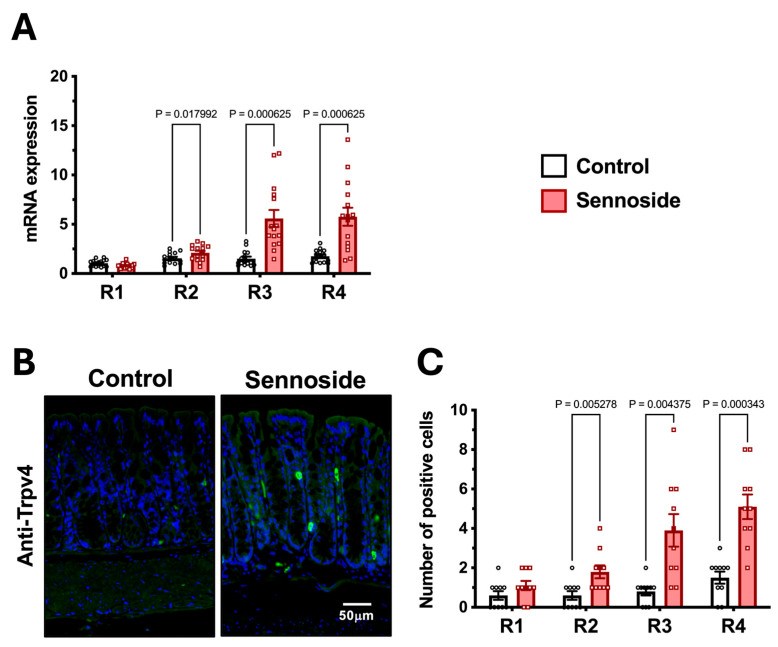
Induction of TRP channels in the distal colon of sennoside-treated mice. mRNA expression of *Trpv4* was analyzed in four colonic regions on day 21 (**A**). Representative immunohistochemical staining for Trpv4 in region 4, as defined in Figure S4, and quantification of Trpv4-positive cells are shown (**B**,**C**). Data are presented as mean ± standard error of the mean. Each dot represents one mouse. *n* = 15 mice per group. Green, Trpv4; blue, DAPI.

Based on reports that colonic overexpression of TRPV4 is associated with the severity of constipation [19], this study also focused on Trpv4 for investigation. The expression of mechanosensitive TRP channels was analyzed to evaluate physicochemical responses to sennoside administration. *Trpv4* mRNA expression increased progressively toward the distal colon, with the highest levels detected in the rectal region following sennoside treatment (Figure 3A).

Immunohistochemical analysis demonstrated that Trpv4 was sparsely distributed within the mucosal epithelium rather than uniformly expressed across epithelial cells (Figure 3B). Trpv4-positive cells were infrequently detected in control tissues but increased in both number and staining intensity following sennoside administration (Figure 3C). This increase was more prominent in distal colonic regions, consistent with the mRNA expression profile (Figure 3C).

### 2.4. E-Cadherin Redistribution and Enhanced Junctional Signaling

**Figure 4:**
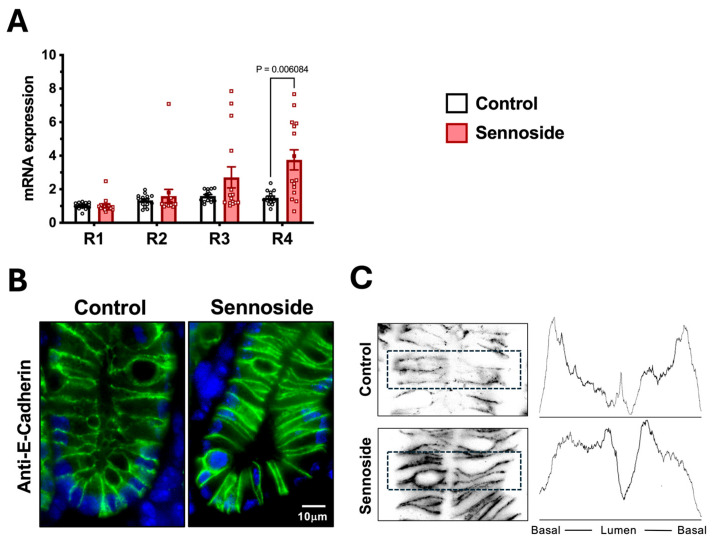
E-cadherin expression and localization in colonic tissues of sennoside-treated mice. *Cdh1* mRNA expression was analyzed in four colonic regions on day 21 (**A**). Representative immunohistochemical staining for E-cadherin in region 4, as defined in Figure S4, and densitometric analysis of staining intensity are shown (**B**,**C**). Data are presented as mean ± standard error of the mean. Each dot represents one mouse. *n* = 15 mice per group. Green, E-cadherin; blue, DAPI.

Intercellular adhesion molecules have been reported to sense mechanical tension and mechanically couple adjacent epithelial cells [20,21]. We therefore focused on E-cadherin, a core adherens junction protein constitutively expressed in the intestinal epithelium, and analyzed its expression and localization. *E-cadherin (Cdh1)* mRNA expression increased progressively toward the distal colon following sennoside administration, with the highest levels detected in the rectal region (Figure 4A). This regional distribution paralleled that of *Trpv4*.

Immunohistochemical analysis demonstrated that, in control tissues, E-cadherin staining was predominantly localized along the basolateral membrane. In contrast, in sennoside-treated tissues, E-cadherin staining intensity increased and extended beyond the basolateral membrane toward the luminal surface (Figure 4B,C). Thus, sennoside administration was associated with both increased E-cadherin expression and altered subcellular distribution.

### 2.5. Mechanical Stress–Induced Cell Spreading

Based on the in vivo findings shown in Figure 2, Figure 3 and Figure 4, in vitro experiments were performed using colonic epithelial cells. CT26 murine colon carcinoma cells were treated with sennoside, and cell proliferation was assessed to determine an appropriate non-cytotoxic concentration for subsequent mechanostimulation experiments. Cell viability was evaluated after 3 days of treatment with low and high concentrations of sennoside (10 ng/mL and 1 μg/mL, respectively). No statistically significant or concentration-dependent differences in proliferation were observed between groups (Figure S7). Therefore, subsequent in vitro experiments were performed using sennoside at 1 μg/mL under non-cytotoxic conditions to evaluate epithelial mechanotransduction and junctional remodeling.

**Figure 5:**
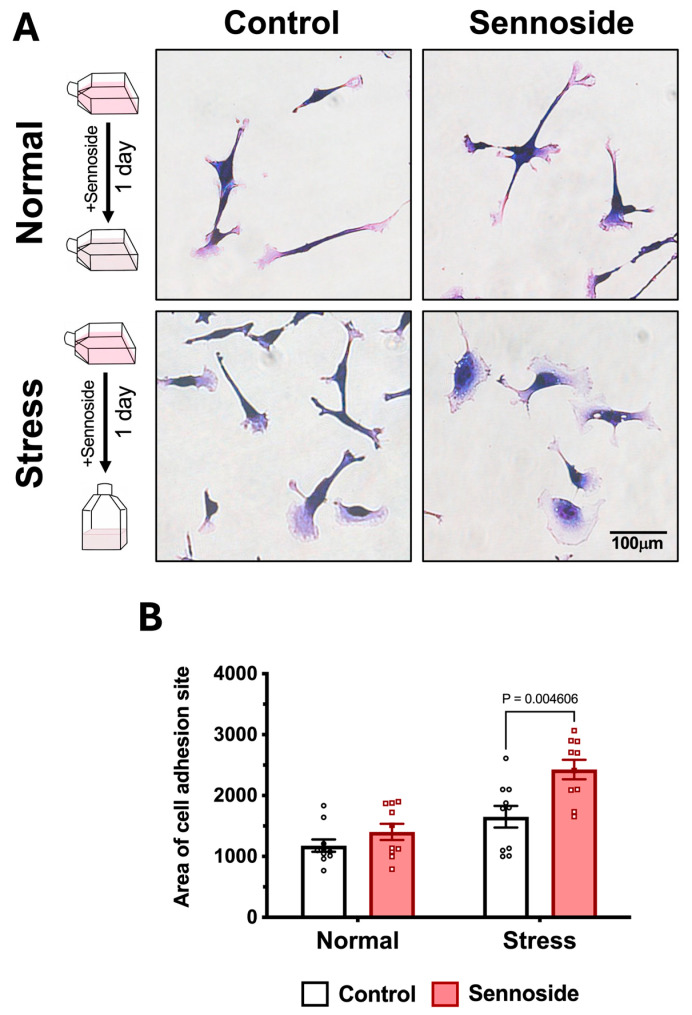
Effects of sennoside on epithelial cell morphology under gravitational stress. CT26 cells were cultured under standard or inverted (gravitational loading) conditions in the presence or absence of sennoside. Representative May–Giemsa–stained images are shown (**A**). Adhesion areas were quantified (**B**). Data are presented as mean ± standard error of the mean. Each dot represents one independent experiment. Experiments were performed in triplicate.

TRP channels contribute to visceral mechanosensitivity and are activated in response to membrane stretch and changes in cell volume [22,23]. Because *Trpv4* and *Trpm8* expression were altered in colonic tissues in vivo, cultured cells were subjected to inverted culture conditions to impose gravitational load. Under gravitational load, morphological changes were observed even in the absence of sennoside, with cells transitioning from a filopodia-like morphology to a lamellipodia-like morphology with an expanded adhesion area (Figure 5A). These morphological changes were more pronounced in the presence of sennoside.

Quantitative analysis demonstrated no significant change in cell length (Figure S8), whereas the adhesion area increased following sennoside treatment (Figure 5B and Figure S8).

### 2.6. Mechanical Stress-Associated TRP Upregulation and Barrier Enhancement

**Figure 6:**
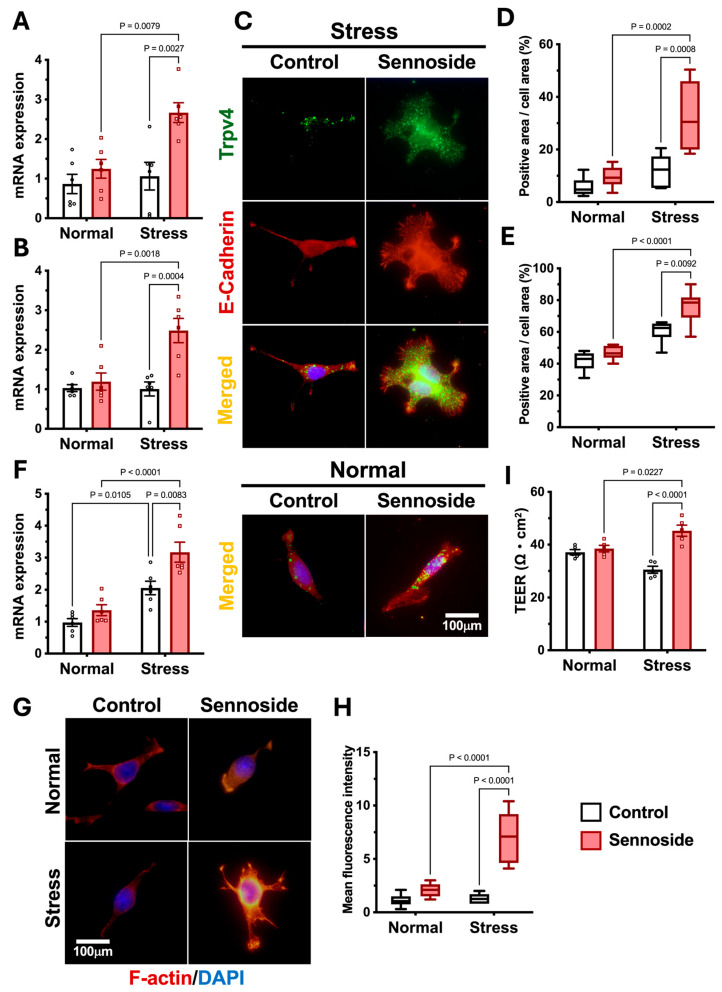
Effects of sennoside on TRP channels, E-cadherin, mechanotransduction-associated responses, and barrier function under gravitational loading. mRNA expression levels of *Trpv4* (**A**) and *Cdh1* (**B**) in CT26 cells cultured under standard or gravitational loading conditions are shown. (**C**) Representative immunofluorescent images of Trpv4 and E-cadherin. The Trpv4-positive area (**D**) and E-cadherin-positive area (**E**) were quantified as percentages of the total cell area from the images shown in (**C**). mRNA expression levels of *Ccn1* are shown (**F**). F-actin organization was evaluated by phalloidin staining (**G**), and the mean fluorescence intensity of F-actin was quantified (**H**). Transepithelial electrical resistance (TEER) was measured using cell culture inserts (**I**). Data are presented as mean ± standard error of the mean. Each dot represents one independent experiment. Experiments were performed in triplicate. In (**C**), green indicates Trpv4, red indicates E-cadherin, and blue indicates DAPI. In (**G**), red indicates F-actin and blue indicates DAPI.

The expression level of *Trpv4* was examined in epithelial cells under mechanical loading. Under standard culture conditions, sennoside treatment increased *Trpv4* expression. This increase was further augmented under inverted culture conditions (Figure 6A). *E-cadherin (Cdh1)* expression showed a similar pattern (Figure 6B).

Immunofluorescence analysis demonstrated that Trpv4 was detected in punctate cytoplasmic clusters, predominantly localized in the perinuclear region (Figure 6C). Under gravitational loading combined with sennoside treatment, Trpv4 staining intensity increased and extended toward cellular protrusions and peripheral regions. E-cadherin protein expression also increased and displayed fibrous localization at adhesion sites (Figure 6C). Quantification of the Trpv4-positive area and E-cadherin-positive area, expressed as percentages of the total cell area, showed trends consistent with the corresponding mRNA expression patterns (Figure 6D,E).

To examine mechanotransduction-associated responses under gravitational loading, we next evaluated *Ccn1* mRNA expression and F-actin organization. *Ccn1* mRNA expression showed a pattern like that of *Trpv4* and *Cdh1* under sennoside-treated gravitational loading conditions (Figure 6F). In addition, Ccn1 expression was increased by gravitational loading alone compared with standard control conditions (Figure 6F). F-actin staining with phalloidin showed a similar tendency to Trpv4 and E-cadherin (Figure 6G), with increased F-actin fluorescence intensity under gravitational loading combined with sennoside treatment (Figure 6H).

Measurement of TEER revealed increased resistance values in sennoside-treated cells under gravitational loading compared with control conditions (Figure 6I).

### 2.7. TRPV4 Dependence of Adhesion and Barrier Responses

**Figure 7:**
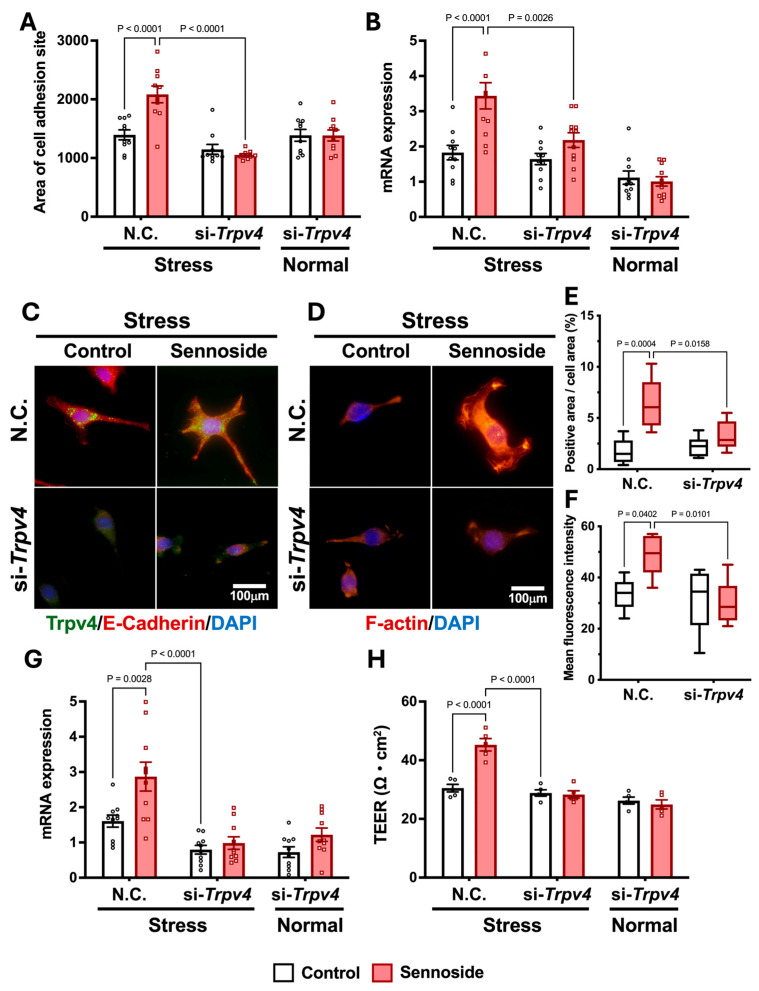
Trpv4 dependence of adhesion, cytoskeletal organization, mechanotransduction-associated responses, and barrier function under gravitational loading. CT26 cells transfected with *Trpv4* or negative control siRNA were cultured under standard or gravitational loading conditions in the presence of sennoside. Adhesion area (**A**), *E-cadherin (Cdh1)* mRNA expression (**B**), representative immunostaining for E-cadherin (**C**), and phalloidin staining for F-actin (**D**) are shown. The E-cadherin-positive area was quantified from the images shown in (**C**) and expressed as a percentage of the total cell area (**E**). The mean fluorescence intensity of F-actin was quantified from the phalloidin-stained images (**F**). *Ccn1* mRNA expression (**G**) and transepithelial electrical resistance (TEER) measurements (**H**) are shown. Data are presented as mean ± standard error of the mean. Each dot represents one independent experiment. Experiments were performed in triplicate. N.C.: negative control siRNA.

To determine the contribution of Trpv4 to epithelial adhesion, cytoskeletal organization, mechanotransduction-associated responses, and barrier function, *Trpv4* expression was suppressed using siRNA. *Trpv4* knockdown attenuated the expansion of the adhesion area observed under combined gravitational loading and sennoside treatment (Figure 7A). *E-cadherin (Cdh1)* expression was reduced in *Trpv4* siRNA–treated cells (Figure 7B). Consistently, E-cadherin immunostaining showed a reduced E-cadherin-positive area after *Trpv4* knockdown (Figure 7C,E). F-actin staining with phalloidin also showed reduced cytoskeletal organization in *Trpv4* siRNA–treated cells, as supported by decreased mean fluorescence intensity of F-actin (Figure 7D,F). *Ccn1* mRNA expression was also attenuated by *Trpv4* knockdown (Figure 7G). TEER values were reduced to levels comparable to those in control cells (Figure 7H).

## 3. Discussion

In the present study, our findings suggest that intestinal epithelial cells may be involved in local changes in gastrointestinal function through mechanosensitive signaling (mechanotransduction) and remodeling of cell–cell adhesions. Traditionally, the effects of stimulant laxatives have been interpreted primarily in terms of enteric neural activation and smooth muscle contraction. Previous studies have shown that sennoside stimulates colonic motility mainly via activation of enteric neurons and prostaglandin E2-mediated smooth muscle contraction [7,8,24]. This classical mechanism is primarily based on metabolite-dependent activation of neuromuscular pathways. In the present study, we focused on epithelial responses associated with TRPV4 induction, E-cadherin-associated junctional remodeling, and adaptive barrier regulation under mechanical stimulation. Thus, our findings do not replace the established prostaglandin E2-mediated laxative mechanism, but suggest that epithelial mechanoadaptation may be associated with sennoside-induced changes in bowel function.

In this study, we combined in vivo and in vitro analyses to identify the induction of TRP family channels under stimulatory conditions, together with structural and functional changes in epithelial adhesion and barrier properties mediated by E-cadherin. Consistent with previous reports demonstrating that TRP channels, including TRPV4, function as mechanosensors in the gastrointestinal tract and are activated in response to membrane stretch, osmotic stress, and cytoskeleton-associated mechanical cues [14,22,23], our findings suggest that epithelial mechanotransduction may be linked to regional motor responses. Furthermore, alterations in E-cadherin-mediated adhesion have been implicated in epithelial barrier remodeling under inflammatory or mechanical stress conditions [16,17,18], supporting the concept that epithelial plasticity modulates intestinal physiology.

Notably, in our experimental model, sennoside administration selectively increased stool water content and distal colonic motility without affecting food intake or body weight. Although earlier studies emphasized secretory mechanisms and altered fluid transport in laxative-induced stool softening [9,25], our data indicate that region-specific alterations in distal colonic motor control also contribute to increased fecal water content. Increased fecal water content was evident as early as day 7 and temporally and spatially coincided with enhanced spontaneous contractions and cholinergic sensitivity in the rectum. These findings suggest that sennoside-associated changes in fecal properties may not be explained solely by osmotic effects on luminal contents but may also involve region-specific changes in distal colonic motor control.

### 3.1. Activation of Epithelial Mechanosensitive Ion Channels

TRP channels are multimodal sensors that convert thermal, mechanical, osmotic, and chemical stimuli into electrochemical signals and are widely expressed in gastrointestinal epithelia and neurons [14,22,26]. Among these channels, TRPV4 responds to membrane stretch, osmotic changes, and physiological temperature ranges (approximately 27–35 °C) and has been implicated in epithelial barrier homeostasis and intestinal mechanosensitivity [14,22,26,27]. Clinically, excessive TRPV4 expression in the colon correlates with constipation severity [20]. In this context, induction of TRPV4 in the distal colon and the accompanying enhancement of rectal motility observed in this study are consistent with the possibility that epithelial TRPV4-associated mechanotransduction is linked to sennoside-induced changes in distal colonic function [14,22,23].

Importantly, TRPV4 induction was not uniform throughout the colon but progressively increased toward distal segments, with the highest expression in the rectum. This spatial specificity is consistent with the anatomical and functional characteristics of the distal colon, which is exposed to greater luminal pressure and tensile stress during stool storage and expulsion. Therefore, TRP channel induction by sennoside may reflect a localized adaptive response to region-specific mechanical demands, although a pharmacologic contribution cannot be excluded. This interpretation is further supported by concordant enhancement of spontaneous motility and cholinergic responsiveness in the same distal regions.

Beyond the TRP family, mechanosensitive ion channels such as Piezo1 and Piezo2 have gained attention. Activation of Piezo1 in the intestinal epithelium regulates cell stretching, regeneration, and mucus secretion [15,28]. TRPV4 and Piezo channels have been implicated in cytoskeleton-associated mechanotransduction pathways [29], suggesting that TRPV4-associated responses observed after sennoside treatment may be part of a broader mechanotransduction network related to intestinal motility.

### 3.2. E-Cadherin-Mediated Adhesion and Barrier Remodeling

E-cadherin is a core component of epithelial adhesion complexes and functions as a mechanosensitive molecule that regulates cell polarity, adhesion strength, and barrier integrity via the cadherin–catenin complex [17,18]. In the present study, sennoside administration increased E-cadherin expression and expanded its localization from the basal membrane toward the luminal surface. These changes indicate reinforcement of intercellular adhesion and epithelial polarity remodeling [16,18,30], consistent with altered barrier-related properties. Correspondingly, in vitro experiments demonstrated increased TEER and augmented cell spreading, supporting the interpretation that sennoside treatment is associated with enhanced electrical and mechanical epithelial barrier properties.

A notable methodological feature of this study is the use of an inverted culture system as a simplified in vitro approach to apply sustained physical stimulation without artificial stretching. This system was not intended to reproduce the complex mechanical environment of the intestine, but rather to examine whether epithelial cells can respond to sustained physical loading under controlled conditions. In the revised experiments, gravitational loading increased Ccn1 expression, a representative YAP/TAZ-associated mechanotransduction target gene [31,32], supporting the interpretation that this condition elicited a mechanotransduction-associated transcriptional response. Under these conditions, TRPV4 induction was accompanied by enhanced cell adhesion, and sennoside further augmented these epithelial responses. Because actin cytoskeletal remodeling is closely linked to YAP/TAZ-mediated mechanotransduction [32], F-actin staining was also used to evaluate cytoskeletal reorganization under sennoside-treated gravitational loading conditions. TRPV4 protein levels increased with sennoside treatment, with redistribution toward cellular protrusions and peripheral regions associated with adhesion and spreading. The appearance of finely dispersed TRPV4 clusters may reflect altered localization of TRPV4 at mechanically responsive cellular interfaces.

Consistently, Trpv4 knockdown significantly attenuated cell spreading, reduced E-cadherin expression, decreased F-actin fluorescence intensity, attenuated Ccn1 expression, and restored TEER values to control levels. These findings suggest that TRPV4 may be involved in E-cadherin–centered adhesion control and epithelial barrier homeostasis. This interpretation is supported by previous studies identifying TRPV4 as a regulator of junctional dynamics and mechanosensitive signaling, as well as reports highlighting the role of E-cadherin–based adhesion in integrating mechanical tension and barrier function [17,20,27,29].

Although reduced Muc2 expression and a trend toward fewer Ki-67-positive cells were observed, these findings should be interpreted cautiously, as they may reflect changes in mucus-related barrier function and epithelial turnover [28,30]. Together with the increase in E-cadherin expression and TEER, these changes may indicate epithelial remodeling associated with altered barrier-related properties [17,20]. However, TEER alone does not fully define epithelial barrier function, and permeability assays or tight junction protein analyses were not performed. Therefore, the TEER findings should be interpreted as increased epithelial electrical resistance rather than definitive evidence of globally enhanced barrier integrity. The decrease in *Muc2* expression further suggests that the barrier response reflects epithelial remodeling rather than uniform barrier enhancement.

Because additional tissue integrity assays, detailed inflammatory histology, and comprehensive immunological profiling were not performed, epithelial stress or early mucosal alteration cannot be excluded. Therefore, the reduced Muc2 and Ki-67 signals may be interpreted as part of epithelial remodeling under the present experimental conditions, but should not be regarded as definitive evidence of physiological adaptation. Temporary thinning of the mucosal layer may increase epithelial exposure to luminal mechanical cues and potentially facilitate TRP-mediated mechanosensing [22,23], consistent with dynamic regulation of epithelial function under mucosal or mechanical conditions [30].

### 3.3. Epithelial–Neural–Muscular Crosstalk in the Distal Colon

The distal colon and rectum are involved in stool storage and defecation, where luminal distension and mechanical forces are likely to be increased [33,34]. Under such conditions, intestinal epithelial cells may sense mechanical cues and release mediators such as adenosine triphosphate and serotonin (5-hydroxytryptamine), which have been reported to influence submucosal and enteric neural circuits [14,23,28,30].

In the present study, TRPV4 induction was most evident in the distal colon and rectum, where enhanced spontaneous motility and increased cholinergic responsiveness were also observed. To assess cholinergic sensitivity more specifically, oxotremorine was used instead of carbachol. Unlike carbachol, which activates both muscarinic and nicotinic receptors, oxotremorine acts as a muscarinic receptor agonist, allowing assessment of muscarinic receptor–dependent responses associated with distal colonic motility [35].

These findings suggest a possible association between distal epithelial mechanosensitive responses and altered colonic motor function. However, the present data do not directly demonstrate neural activation, mediator release, or causal transmission from epithelial TRPV4 signaling to smooth muscle contraction. Therefore, TRPV4-associated epithelial mechanotransduction should be interpreted as an epithelial response that accompanies enhanced distal colonic motility, rather than as direct evidence of epithelial-to-neuromuscular signaling.

Taken together, these findings support the possibility that region-specific mechanical environments may influence epithelial mechanosensitivity in the distal colon. Further studies are required to determine whether these epithelial changes contribute directly to downstream neuromuscular regulation.

### 3.4. Improvement of Bowel Function Through Noninflammatory Mechanisms

Sennoside is metabolized by the intestinal microbiota into active compounds such as sennidin and rheinanthrone, which are believed to mediate its classical pharmacologic effects [9,24]. However, the epithelial mechanisms associated with sennoside treatment remain unclear. Although rheinanthrone is an important active metabolite, the present in vitro findings showed that epithelial responses to sennoside were observed under mechanical stress conditions. These observations do not exclude the established contribution of microbial metabolites such as rheinanthrone in vivo but raise the possibility that epithelial responses to the parent compound may also be involved in the context of luminal mechanical stimulation. The relative contributions of sennoside and its metabolites should be further examined in future studies.

Although long-term use of stimulant laxatives has historically been associated with epithelial atrophy, melanosis coli, and inflammatory or oxidative mechanisms, recent systematic reviews and epidemiologic studies indicate no clear increase in colorectal cancer risk with appropriate stimulant laxative use [11,12,13]. The present findings may provide a possible mechanistic perspective on this safety profile: sennoside was associated with mechanical and adhesive remodeling without provoking inflammatory responses. The absence of changes in canonical inflammatory cytokines, such as IL-1β and IL-6, supports the conclusion that sennoside occurred without overt induction of these inflammatory markers.

Conversely, multiple large-scale cohort studies have demonstrated that chronic constipation is associated with significantly reduced long-term survival (>15 years) [4], indicating that untreated constipation is not benign. The epithelial mechanosensing–related changes observed in this study may provide a physiological perspective for understanding bowel function improvement, although their relevance to long-term constipation treatment requires further investigation.

### 3.5. Clinical Implications and Limitations

This study suggests that epithelial plasticity may be considered as an additional component of stimulant laxative-associated bowel function, alongside the traditional neuromuscular activation model. Based on these findings, potential future directions include therapeutic strategies targeting the TRPV4–E-cadherin axis, the development of distal colon–selective formulations, and dosing approaches that consider the temporal dynamics of epithelial responses.

However, several limitations should be acknowledged. Although additional experiments were performed to increase the robustness of the motility assays, no a priori power calculation was conducted. Therefore, the overall findings should be interpreted with appropriate caution. Although the use of healthy mice does not fully recapitulate the pathological features of chronic constipation, this model is suitable for investigating fundamental regulatory mechanisms of intestinal function. The generalizability of this mechanism should be further evaluated in other models, including opioid-induced and loperamide-induced constipation [36,37]. Importantly, in vivo causality between epithelial TRPV4 activation and enhanced distal colonic motility remains to be established. In addition, TRPV4 expression appeared to be localized to specific epithelial subtypes; however, precise identification of these cell populations was not achieved. Although the primary purpose of the immunofluorescence analyses was to visualize the localization patterns of E-cadherin and TRPV4, fluorescence intensity was additionally quantified to support these observations. However, complementary protein-level validation by Western blotting or quantitative proteomic analysis was not performed. Furthermore, the in vitro experiments have important limitations. CT26 cells are tumor-derived and do not fully reflect normal intestinal epithelial physiology, and the gravity-loading model based on flask inversion is a simplified approach that does not reproduce the complex biomechanical environment of the intestine. Therefore, these findings should be interpreted as supportive mechanistic evidence, and further validation using primary epithelial cells, intestinal organoids, or ex vivo tissue-based mechanical loading systems will be required. The relative contributions of sennoside and its metabolites, including rheinanthrone, should also be further examined in future studies.

## 4. Materials and Methods

### 4.1. Animals

Five-week-old male BALB/cAJcl mice were obtained from CLEA Japan Inc. (Tokyo, Japan). Animals were randomly assigned using a simple random allocation method to either a control (vehicle-treated) group or a sennoside-treated group. This study compared sennoside-treated mice with control mice; therefore, no additional control groups were included. Although the number of animals varied depending on the experimental endpoint, 8–15 animals were allocated to each experimental group at each time point. Sample sizes were determined based on prior studies and preliminary experiments evaluating intestinal motility and molecular endpoints to ensure adequate biological replication. The sample size was considered sufficient to detect biologically relevant differences based on prior experience. Because multiple region-specific and mechanistic outcomes were assessed, a formal a priori power calculation was not performed. No animals or data points were excluded from the analysis, and no predefined exclusion criteria were established. Outcome assessment was not fully blinded across all experiments. However, for the additional motility assays performed during revision, outcome assessment was conducted under blinded conditions where feasible. This study was conducted in accordance with the ARRIVE guidelines, and details of ethical approval are described in the Institutional Review Board Statement section.

Mice were maintained in a specific pathogen–free (SPF) facility at the School of Allied Health Sciences, Kitasato University, under controlled environmental conditions (23 ± 3 °C; 12 h light/dark cycle). Animals had ad libitum access to water and were fed a standard commercial diet (CE-2; CLEA Japan, Inc.). Sennoside (Alfresa Pharma Corp., Osaka, Japan), a preparation containing a mixture of sennosides A and B with a total sennoside content of 8%, was dissolved in 0.5% (*w*/*v*) carboxymethyl cellulose (CMC). The administered dose was calculated based on the actual sennoside content, and sennoside was administered once daily via oral gavage using a gastric feeding needle at a dose equivalent to 4.8 mg/kg body weight for 21 consecutive days. The dose of 4.8 mg/kg body weight was selected because it corresponds to a human equivalent dose of approximately 0.39 mg/kg based on body surface area conversion, or approximately 23 mg/day for a 60 kg adult, which is close to the upper range of the usual therapeutic dose of sennoside in adults. Administration was performed at a consistent time each day to minimize circadian variability.

Mice were housed in groups of 3–4 per standard bedding cage. Bedding was changed at least once weekly. Metabolic cages were not used. Body weight was recorded daily. Animals were monitored daily for general health status, and no adverse events were observed. Food intake and water consumption were measured per cage by weighing the remaining food and water and were expressed as the mean intake per mouse by dividing total consumption by the number of animals per cage. At the experimental endpoint, mice were euthanized by cervical dislocation, and the colon was immediately excised for downstream analyses. All experimental procedures were performed under the supervision of investigators experienced in animal handling.

### 4.2. Fecal Observation and Analysis

To minimize circadian variability, fecal samples were collected at approximately 10:00. The number and total weight of fecal pellets were measured and expressed per hour. Fecal water content was determined using feces collected from the rectum at the time of dissection and was calculated as the difference between wet weight and dry weight after freeze-drying. Stool consistency was evaluated using the Bristol Stool Form Scale (BSFS) [38], a validated 7-point scoring system ranging from type 1 (hard, separate lumps) to type 7 (watery stool without solid pieces), with higher scores indicating softer stool consistency. Although originally developed for clinical assessment in humans, the scale was adapted for murine fecal evaluation based on macroscopic appearance, consistent with its application in experimental rodent studies.

### 4.3. Gene Expression Analysis

Total RNA was extracted from colonic tissues using TRIzol reagent (Thermo Fisher Scientific Inc., Waltham, MA, USA) according to the manufacturer’s instructions. Complementary DNA (cDNA) was synthesized from total RNA by reverse transcription using the PrimeScript RT Reagent Kit (Takara Bio Inc., Shiga, Japan). Real-time PCR was performed using SYBR Select Master Mix (Thermo Fisher Scientific Inc.) to amplify transcripts encoding *Muc1*, *Muc2*, *Muc5ac*, *Il1b*, *Il4*, *Il6*, *Il10*, *Tnfα*, *Trpv4*, *Trpm8*, *Cdh1* (E-cadherin), *Ccn1* and *Gapdh*. Amplification was carried out using the ABI 7500 Real-Time PCR System (Applied Biosystems, Foster City, CA, USA). The thermal cycling conditions were as follows: initial denaturation at 95 °C for 10 min, followed by 40 cycles of denaturation at 95 °C for 15 s and annealing/extension at 60 °C for 1 min. A melting curve analysis was performed to confirm amplification specificity.

Relative gene expression levels were calculated using the comparative Ct (2^−ΔΔCt^) method and normalized to *Gapdh* as the internal control. Primer sequences were as follows:

- *Muc1*: 5′-GTCTTCAGGAGCTCTGGTGG and 5′-TACCACTCCAGTCCACAGCA
- *Muc2*: 5′-GCTGACGAGTGGTTGGTGAATG and 5′-GATGAGGTGGCAGACAGGAGAC
- *Muc5ac*: 5′-CATGGAGGGGACCTGGAAAC and 5′-CCACATGGGGTCACACTTC
- *Il1β*: 5′-TGGACCTTCCAGGATGAGGACA and 5′-GTTCATCTCGGAGCCTGTAGTG
- *Il4*: 5′-ATCATCGGCATTTTGAACGAGG and 5′-ACCTTGGAAGCCCTACAGACGA
- *Il6*: 5′-TACCACTTCACAAGTCGGAGGC and 5′-CTGCAAGTGCATCATCGTTGTTC
- *Il10*: 5′-CGGGAAGACAATAACTGCACCC and 5′-CGGTTAGCAGTATGTTGTCCAGC
- *Tnfα*: 5′-GGTGCCTATGTCTCAGCCTCTT and 5′-GCCATAGAACTGATGAGAGGGAG
- *Trpv4*: 5′-TCACCGCCTACTATCAGCCACT and 5′-GAACAGGACTCCTGTGAAGAGC
- *Cdh1* (E-cadherin): 5′-CTACAGCATCACCGGCCAA and 5′-ACACGGCATGAGAATAGAGGATG
- *Gapdh*: 5′-AACTTTGGCATTGTTGTGGAAGG and 5′-ACACATTGGGGGTAGGAACA
- *Ccn1*: 5′-GTGAAGTGCGTCCTTGTGGACA and 5′- CTTGACACTGGAGCATCCTGCA

### 4.4. Histopathological Analysis

Excised colonic tissues were fixed for 24 h in freshly prepared 4% paraformaldehyde in phosphate-buffered saline (PBS), rinsed with PBS, and embedded in paraffin. Sections (4 μm thickness) were deparaffinized in xylene and dehydrated through graded ethanol.

For immunohistochemical staining, sections underwent heat-induced antigen retrieval in 10 mmol/L citrate buffer (pH 6.0) at 95 °C for 10 min, followed by gradual cooling to room temperature (20–25 °C). Sections were permeabilized with 0.5% Triton X-100 in PBS for 10 min at room temperature. For horseradish peroxidase (HRP)/3,3′-diaminobenzidine (DAB) detection, endogenous peroxidase activity was quenched by incubation with 3% H_2_O_2_ in PBS for 10 min at room temperature.

Sections were blocked with Protein Block (Agilent Technologies, Inc., Santa Clara, CA, USA) for 30 min at room temperature. Primary antibodies against Muc2 (Santa Cruz Biotechnology, Dallas, TX, USA; catalog no. sc-15334), Ki-67 (Abcam, Cambridge, UK; catalog no. ab15580), Trpv4 (Abcam; catalog no. ab30744), E-cadherin (R&D Systems, Inc., Minneapolis, MN, USA; catalog no. AF748) and Alexa Fluor 555 Phalloidin (Thermo Fisher Scientific Inc.; catalog no. A34055) were diluted in Antibody Diluent with Background-Reducing Components (Agilent Technologies, Inc.) and incubated overnight at 4 °C.

After washing, sections were incubated for 30 min at room temperature with appropriate antibodies: anti-rabbit IgG-Alexa Fluor 488 (Cell Signaling Technology, Inc., Danvers, MA, USA; catalog no. 4412) or anti-goat IgG-Alexa Fluor 594 (Abcam; catalog no. ab150140) for fluorescence detection. Nuclei were counterstained with DAPI (DOJINDO LABORATORIES, Kumamoto, Japan), and sections were mounted using Fluorescence Mounting Medium (Agilent Technologies, Inc.).

For HRP/DAB detection, EnVision+ System-HRP–Labeled Polymer Anti-Rabbit (Agilent Technologies, Inc.) was used as the secondary reagent, followed by nuclear counterstaining with hematoxylin. Chromogenic signals were developed using the ImmPACT DAB Substrate Kit (Vector Laboratories, Inc., Newark, CA, USA). Sections were dehydrated through graded ethanol, cleared in xylene, and mounted.

Tissue observation and image acquisition were performed using a light microscope (Olympus Corporation, Tokyo, Japan). Quantitative image analysis was performed using ImageJ software version 1.54g (National Institutes of Health, Bethesda, MD, USA).

### 4.5. Intestinal Motility Analysis

After euthanasia, the entire intestinal tract was excised, and the mesentery was carefully removed. Tissues were immediately immersed in physiological saline (Otsuka Pharmaceutical Co. Ltd., Tokushima, Japan) until further processing.

Krebs buffer prewarmed to 37 °C was added to a Magnus chamber connected to a circulating heating system to maintain a constant temperature of approximately 37 °C throughout the experiment. The chamber was continuously aerated using an air pump during recordings.

Following gentle luminal rinsing, both ends of the excised intestinal segment were mounted in the Magnus apparatus under defined resting tension. The upper thread was connected to a force transducer (FD Pickup, Nihon Kohden Co., Ltd., Tokyo, Japan), and changes in contractile activity were recorded using a data acquisition system (PowerLab 2/25, ADInstruments, Lexington, KY, USA) with LabChart 8 software (ADInstruments, Bella Vista, NSW, Australia).

After stabilization of spontaneous motility, acetylcholine (ACh) (Sigma-Aldrich Co. LLC, St. Louis, MO, USA) or oxotremorine (Oxo) (Sigma-Aldrich Co. LLC) was added to achieve a final concentration of 5 μM, and contractile responses were recorded. The Krebs buffer was subsequently replaced, and tissues were washed three times with fresh prewarmed Krebs buffer.

### 4.6. Cell Culture and Gravity Loading

The murine colon carcinoma cell line CT26, originally obtained from the American Type Culture Collection (ATCC, Manassas, VA, USA) on 21 January 2009, was cultured in RPMI-1640 medium (FUJIFILM Wako Pure Chemical Corp., Osaka, Japan) supplemented with 10% fetal bovine serum (FBS) (Biowest, Nuaillé, France). The identity of CT26 cells was authenticated on 20 May 2024, by short tandem repeat (STR) profiling, confirming consistency with the reference profile registered in the ATCC.

After 24 h, sennoside was added to the culture medium at a final concentration of 1 μg/mL, and the cells were incubated for an additional 24 h.

To model mechanical loading, culture flasks were inverted to a 90° position at the time of sennoside addition, thereby altering the direction of gravitational force acting on adherent cells and providing a sustained physical stimulus. Although this approach does not involve active stretching, changes in gravitational load have been reported to influence cell adhesion, cytoskeletal organization, and mechanosensitive signaling pathways. Mechanical stimuli are known to regulate these processes, including activation of TRP channels and other mechanically activated ion channels [17,20,22,39]. Therefore, this simplified approach was used as an in vitro approximation of mechanical loading.

For *Trpv4* knockdown, cells seeded the previous day were transfected with *Trpv4*-specific small interfering RNA (siRNA) (Thermo Fisher Scientific Inc.) using Lipofectamine RNAiMAX (Thermo Fisher Scientific Inc.) in serum-free Opti-MEM I medium (Thermo Fisher Scientific Inc.), according to the manufacturer’s instructions. After 48 h, knockdown efficiency was confirmed by quantitative real-time PCR (qPCR). Negative control siRNA (Thermo Fisher Scientific Inc.) was used as a control.

Cell length and adhesion area were quantified after May–Giemsa staining.

### 4.7. Trans-Epithelial Electrical Resistance

CT26 cells were seeded into permeable insert membranes and cultured for 24 h. Sennoside (1 μg/mL) was then added, and cells were incubated for an additional 24 h. For gravitational loading, insert plates were inverted to a 90° position for 24 h.

Electrodes were placed in the apical and basolateral compartments, and transepithelial electrical resistance (TEER) was measured using a Millicell ERS-2 Electrical Resistance System (Merck & Co., Inc., Rahway, NJ, USA). TEER values were corrected by subtracting blank insert resistance and expressed as Ω·cm^2^. TEER values were corrected by subtracting the resistance of blank inserts and expressed as Ω·cm^2^, calculated as (R_sample − R_blank) × membrane surface area (0.33 cm^2^ for 24-well inserts).

### 4.8. Statistical Analysis

Quantitative data are presented as the mean ± standard error of the mean (SEM). Statistical analyses were performed using GraphPad Prism (version 10.0; GraphPad Software, San Diego, CA, USA). Data distribution normality was assessed using the Shapiro–Wilk test. For comparisons between two groups, Welch’s *t*-test was used for normally distributed data, whereas the Mann–Whitney U test was applied for non-normally distributed data. For comparisons among three or more groups, one-way analysis of variance (ANOVA) followed by Tukey’s multiple comparisons test or the Kruskal–Wallis test followed by Dunn’s multiple comparisons test was used, as appropriate based on data distribution. For experiments involving two independent variables, two-way ANOVA followed by Tukey’s multiple comparisons test was used. Multiple comparisons were adjusted using the post hoc tests described above where applicable. A *p* value < 0.05 was considered statistically significant. Exact *n* values are indicated in the corresponding figure legends.

## 5. Conclusions

Collectively, these findings show that sennoside induces stepwise and region-specific modulation of stool properties, distal colonic motility, TRP channel expression, and epithelial adhesion–barrier remodeling without overt induction of the inflammatory markers examined. These results support the possibility that TRP channel-associated epithelial mechanotransduction is linked to barrier-related remodeling and regional changes in distal colonic function, thereby providing an additional perspective on the mechanisms of action of stimulant laxatives.

## Data Availability

All data generated or analyzed during this study are included in this published article and its Supplementary Information files. This study was not preregistered. Additional data are available from the corresponding author upon reasonable request.

## Supplementary Materials

The following supporting information can be downloaded at: https://www.mdpi.com/article/10.3390/ijms27115057/s1.

## Abbreviations

The following abbreviations are used in this manuscript:

ACh	acetylcholine
ANOVA	analysis of variance
B.W.	body weight
BSFS	Bristol Stool Form Scale
Ccn1	Cellular Communication Network Factor 1
CMC	carboxymethyl cellulose
cDNA	complementary DNA
Cdh1	E-cadherin
CT26	murine colon carcinoma cell line CT26
DAB	3,3′-diaminobenzidine
DAPI	4′,6-diamidino-2-phenylindole
DMSO	dimethyl sulfoxide
FBS	fetal bovine serum
GI	gastrointestinal
GAPDH	glyceraldehyde-3-phosphate dehydrogenase
HRP	horseradish peroxidase
IHC	immunohistochemistry
IL	interleukin
mRNA	messenger RNA
MTT	3-(4,5-dimethylthiazol-2-yl)-2,5-diphenyltetrazolium bromide
NC	negative control
Oxo	oxotremorine
PBS	phosphate-buffered saline
R1–R4	colonic regions 1–4 (oral→anal)
RPMI	Roswell Park Memorial Institute medium 1640
RT	room temperature
RT-PCR	reverse transcription PCR
SEM	standard error of the mean
siRNA	small interfering RNA
SPF	specific pathogen-free
TEER	transepithelial electrical resistance
TRP	transient receptor potential
TRPV4	transient receptor potential vanilloid 4
