# Supplementary material for: TRPV4-Dependent Epithelial Mechanoadaptation and Barrier Remodeling Mediate Sennoside-Induced Distal Colonic Motility
Source: Int J Mol Sci. 2026 Jun 3;27(11):5057. doi: 10.3390/ijms27115057 (PMC13257419; doi:10.3390/ijms27115057)
Supplement: Supplementary file 1 [file ijms-27-05057-s001.zip › ijms-4300304-supplementary.pdf]

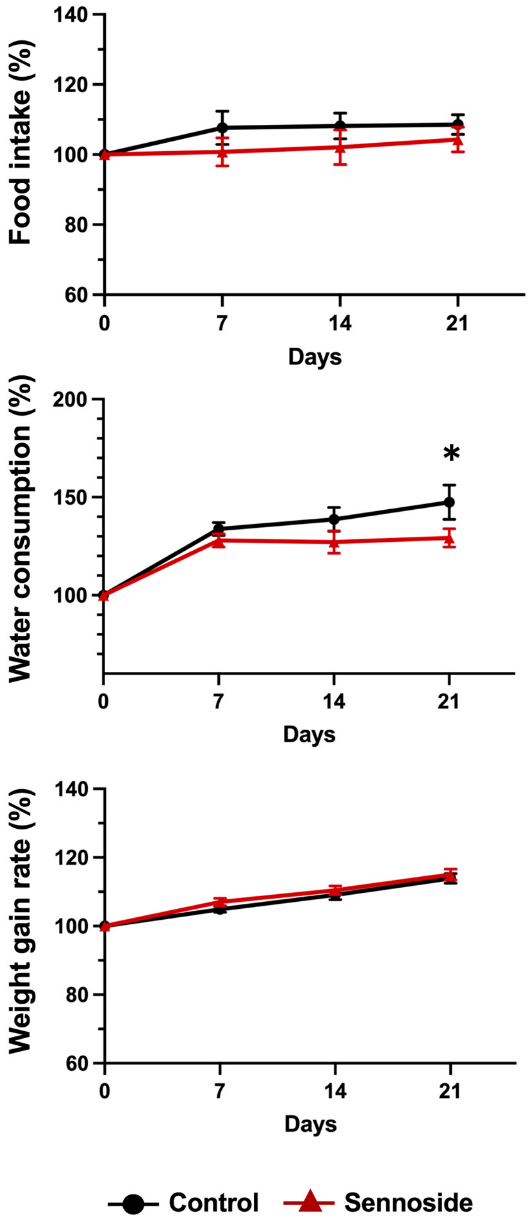

Figure S1. Physiological parameters in sennoside-treated mice. Daily food intake, water consumption, and body weight were measured weekly for 21 days following sennoside administration. Data are presented as mean ± SEM. Each dot represents one mouse. *n* = 15 mice per group. *$p < 0.05$.

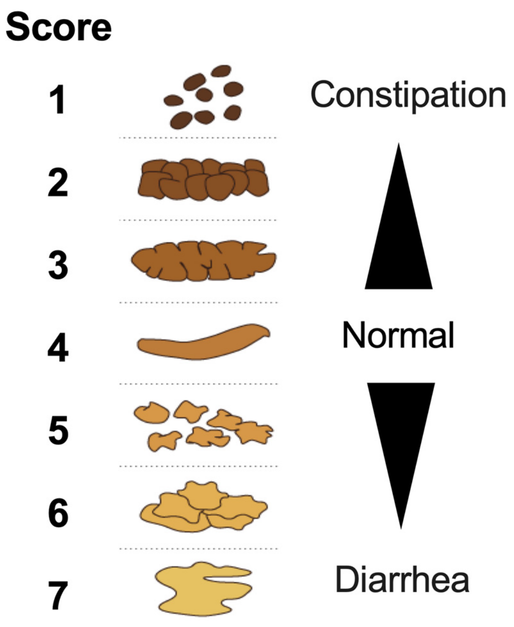

Figure S2. Bristol Stool Form Scale (BSFS). Reference chart used to score stool consistency.

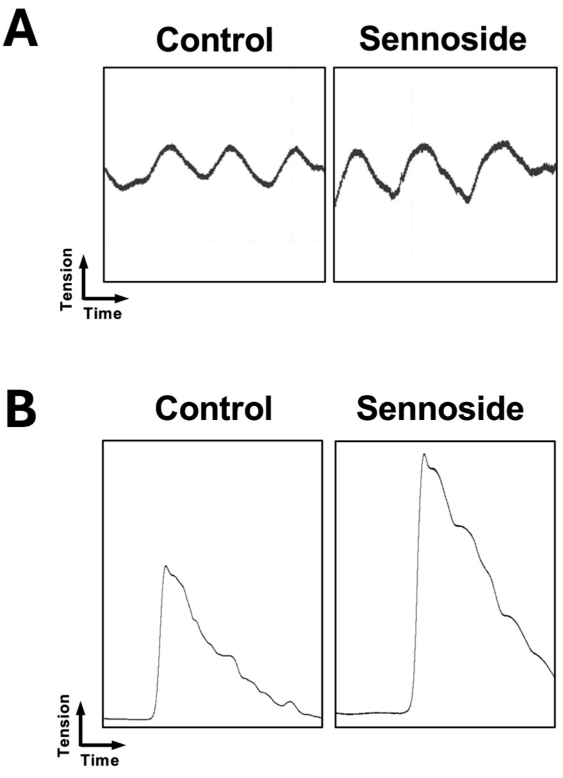

Figure S3. Representative colonic contractile waveforms. Spontaneous contractile activity recorded using the Magnus method is shown in (A). A representative waveform following stimulation with acetylcholine (ACh) is shown in (B). Oxotremorine (Oxo) induced a comparable contractile response.

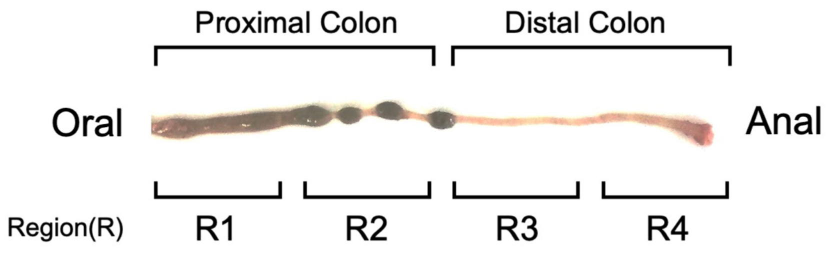

Figure S4. Colonic regions analyzed. Schematic depiction of the four colonic regions (R1–R4) used for region-specific analyses.

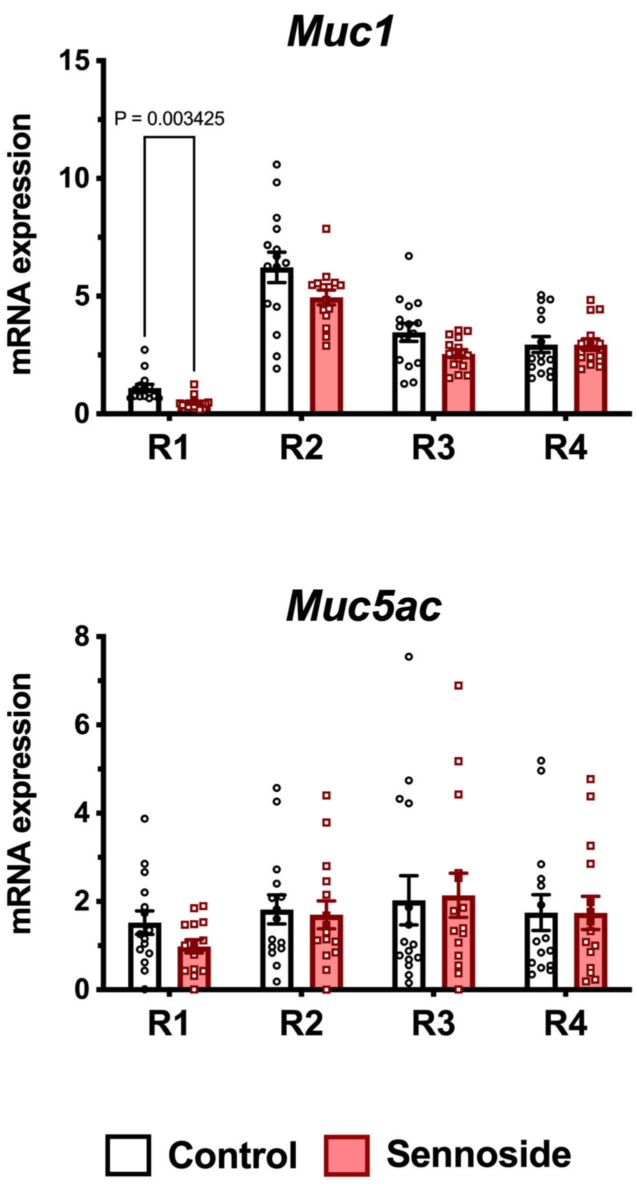

Figure S5. *Muc1* and *Muc5ac* expression in colonic tissues. mRNA expression of *Muc1* and *Muc5ac* in the four colonic regions 21 days after sennoside administration. Data are presented as mean ± SEM. Each dot represents one mouse. n = 15 mice per group.

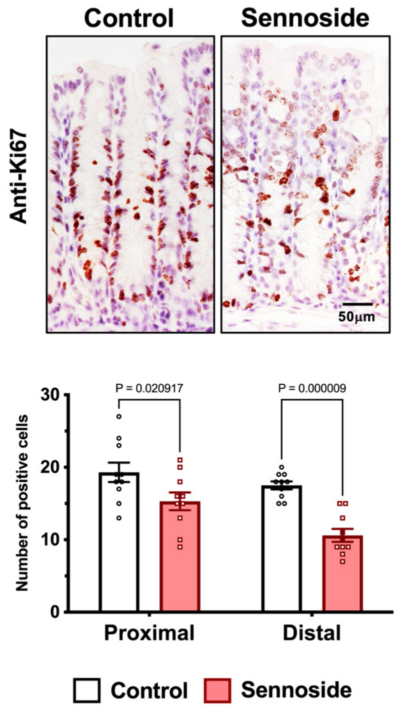

Figure S6. Detection of proliferating cells in colonic tissues. Representative Ki-67 immunostaining and quantification of positive areas. Data are presented as mean ± SEM. Each dot represents one mouse. n = 6–10 mice per group.

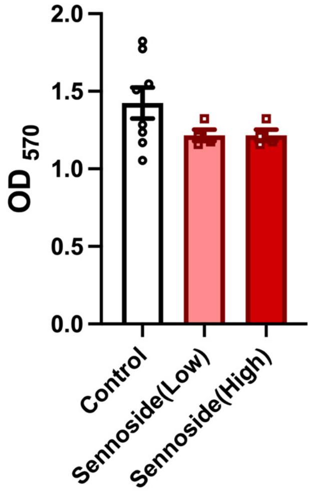

Figure S7. Effects of sennoside on CT26 cell proliferation. Cell viability was assessed using the MTT assay after 3 days of sennoside treatment at low (10 ng/mL) and high (1 μg/mL) concentrations. Data are presented as mean ± SEM. Each dot represents an independent experiment. Experiments were performed in triplicate.

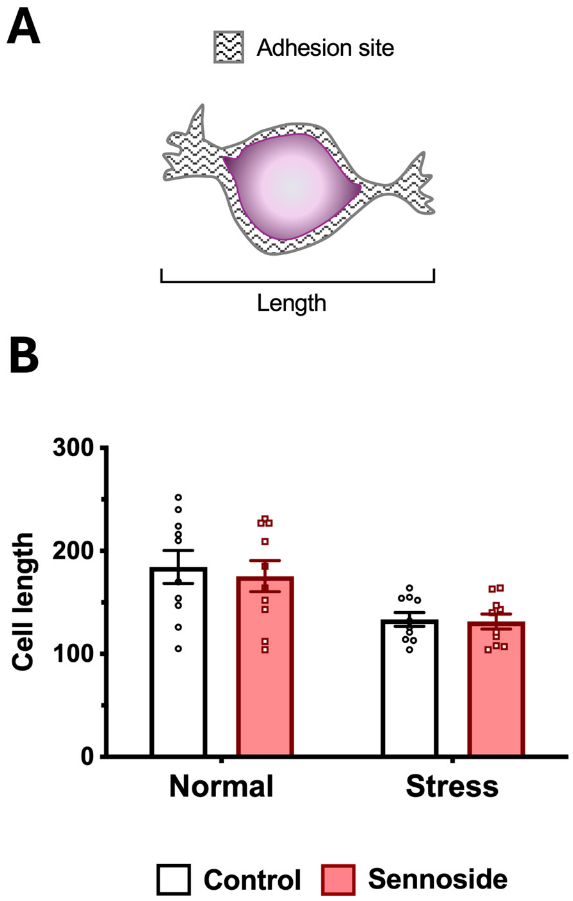

Figure S8. Measurement criteria for cell length and adhesion area. Schematic illustrating the measurement criteria for cell length and adhesion area (A) and comparison of cell lengths across conditions (B). Data are presented as mean ± SEM. Each dot represents an independent experiment. Experiments were performed in triplicate.
